# The Relation of Copper and Zinc with Incidence of Neonatal Sepsis and Possible Prediction Biomarker Role

**DOI:** 10.31661/gmj.v9i0.1933

**Published:** 2020-05-22

**Authors:** Seyed Hossein Saadat, Rakhshaneh Goodarzi

**Affiliations:** ^1^Neonatologist, Clinical Research Development Center of Children Hospital, Hormozgan University of Medical Sciences, Bandar Abbas, Iran

**Keywords:** Copper, Zinc, Neonate, Sepsis

## Dear Editor,

**Figure 1:**
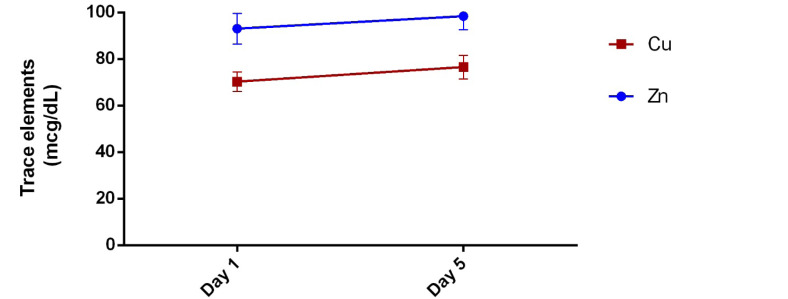
The Mean S.E.M of the Zn and Cu serum levels of the neonate with sepsis on day 1 and 5.

According to recent studies [1,2], nutrition is believed to play a role in the pathogenesis of adverse pregnancy outcomes, including preterm birth and subsequent neonatal sepsis. Trace elements such as copper (Cu) and zinc (Zn) are essential for a pregnancy with good outcomes for both mother and infant. As a result, deficiency of these elements plays an important role in neonatal complications such as neonatal sepsis [3]. In other words, pregnant women, especially in developing countries, are considered a high-risk group for trace elements deficiency, especially Zn and Cu [4]. Previous studies have investigated the association between nutritional deficiencies with the risk of increased neonatal sepsis, but few studies have examined the status of these elements during neonatal sepsis and its association with disease severity [3,4]. Therefore, we conducted a pilot study on 40 term neonates with neonatal sepsis at Bandar Abbas City, Iran in 2019 that were diagnosed according to clinical and laboratory criteria, and serum levels of Cu and Zn at day 1 and day 5 were compared. In this study, 27 male and 13 female infants were enrolled. The mean age of neonates was 8.9±8.44 days and the mean gestational age was 38.72±1.07 weeks. As shown in Figure 1, Zn and Cu levels were 93.05±41.42 and 70.15±26.15 mcg/dL on day 1, respectively, while on day 5, Zn and Cu levels were 98.38±37 and 76.5±32.04 mcg/dL, respectively. According to the results of our study, Cu levels in neonates with neonatal sepsis were higher than the normal range (20-70 mcg/dL), which increased with increasing duration of infection, although this increase was not significant (P˃0.05). The results also showed that Zn levels in neonates, although within the normal range (80-120 mcg/dL) [5] and increased after 5 days, not statistically significant(P˃0.05). Neonatal Cu and Zn levels have a direct relationship with maternal serum levels during pregnancy [6]. While it is difficult to assess the prevalence of Zn deficiency, numerous studies have suggested the possibility of mild to moderate Zn deficiency widely in pregnant women. The prevalence of Zn deficiency in developing countries is probably similar to that of iron deficiency [7]. Caulfield *et al* reported that 82% of pregnant women worldwide are likely to have inadequate Zn intake [8]. Severe Zn deficiency, although uncommon, is associated with spontaneous abortion and congenital malformations. While milder forms of deficiency are associated with low birth weight, intrauterine growth restriction and preterm delivery. In addition, mild Zn deficiency may be associated with complications of childbirth, such as premature rupture of the membranes and subsequent neonatal sepsis [9]. However, the underlying mechanisms of this association are unclear. A Cochrane review of Zn supplementation in pregnancy by Mahomed *et al*. Included 17 studies of 9,000 women and their children [10]. According to 13 randomized clinical trials, 6854 women had a slight but significant decrease in preterm delivery. Regarding neonatal morbidities such as neonatal sepsis, respiratory distress syndrome, and intraventricular hemorrhage, there was no difference between the Zn and control groups. Studies have shown that deficiency or excessive amounts of Cu and Zn can be associated with adverse pregnancy outcomes. Excess Zn can suppress the immune response, reducing Cu levels [11,12]. To the best of our knowledge, no study in Iran has examined the serum levels of Cu and Zn in infants with neonatal sepsis. In the study of Wisniewska *et al*. [13], serum Zn and Cu levels in neonates with sepsis were lower than controls. The results showed that sepsis may increase plasma Cu concentration and relative Zn deficiency. In our study, in line with the results of studies in children and adults, serum Cu and Zn concentrations increased on the fifth day compared to the first day of the disease. Although elevated serum Cu levels as an inflammatory marker and Zn deficiency during neonatal sepsis may be used as a predictor in determining the severity of neonatal sepsis, clinical studies with a more sample size are recommended to found the role of trace elements such as Cu and Zn in the incidence and severity of neonatal sepsis as well as the serum Cu and Zn concentrations (or Cu to Zn ratio) as disease biomarker.
